# Transcriptome analysis of near-isogenic line provides novel insights into genes associated with panicle traits regulation in rice

**DOI:** 10.1371/journal.pone.0199077

**Published:** 2018-06-20

**Authors:** Wuhan Zhang, Pingyong Sun, Qiang He, Fu Shu, Huafeng Deng

**Affiliations:** 1 State Key Laboratory of Hybrid Rice, Hunan Hybrid Rice Research Center, Hunan Academy of Agricultural Sciences, Changsha, China; 2 Collaborative Innovation Center of Grain and Oil Crops in South China, Changsha, China; 3 China National Japonica Rice Research and Development Center, Tianjin, China; Institute of Genetics and Developmental Biology Chinese Academy of Sciences, CHINA

## Abstract

Panicle traits in rice impact yield and quality. The *OsGRF4* gene encodes a growth-regulating factor controlling panicle traits, and was recently cloned. Gene expression profiling analysis can be used to study the molecular mechanisms underlying *OsGRF4* regulation. Use of near-isogenic lines (NILs) reduces genetic background noise in omics studies. We compared transcriptome profiling of 7 cm long young panicles of NIL-*Osgrf4* and NIL-*OsGRF4* using RNAs sequence analyses. Eighty differentially expressed genes (DEGs) were identified. Our target gene *OsGRF4* was up-regulated in NIL-*OsGRF4* plants, which is consistent with a previous qPCR analysis. Hierarchical cluster analysis showed *OsGRF4* is tightly clustered with the up-regulated DEG LOC_Os02g47320. Gene Ontology (GO) and KEGG analysis suggested that DEGs were primarily involved in somatic embryogenesis and chitinase activity. Two up-regulated DEGs, LOC_Os04g41680 and LOC_Os04g41620, were significantly enriched in the top 8 GO terms, and were over_represented in term of seed development, and may play key roles in grain shape regulation. The transcription factor *Osmyb1* also exhibited differential expression between NILs, and may be is an important regulator of panicle traits. By searching reported functions of DEGs and by co-localization with previous identified quantitative trait loci (QTL), we determined that the pleiotropic gene *OsGRF4* may also be involve in abiotic stress resistance. This study provides new candidates genes for further understanding the molecular mechanisms underlying rice panicle trait regulation.

## Introduction

Rice is a crucial agronomic crop worldwide, especially in Asians. By the year 2030, a 40% increase in rice production will be necessary to accommodate the rapidly increasing world population [1]. Panicle traits such as grain size, panicle shape, seed shattering, and seed germination, are the main determinants of yields and quality in rice. Recent developments in molecular genetics approaches have led to cloning of many panicle trait genes in rice. Plant hormones such as cytokinin, auxin, and brassinosteroid (BR) play an importance role in rice panicle development. *Grain number 1a* (*Gn1a*) was the first cloned major QTL responsible for grain number in rice. *Gn1a* encodes a cytokinin oxidase/dehydrogenase (*OsCKX2*), which can reduce the content of phytohormone cytokinin. Low expression level of *Gn1a* causes cytokinin accumulation in the inflorescence meristem, which results in increased grain number and yield in rice [2]. *GAD1* encodes a small signal peptide, and loss of function in *GAD1* leads to increases grain number and grain length in cultivated rice. It was speculated that *GAD1* activates *DST* and *CKX2* to degrade the phytohormone cytokinin, and results in reduced grain number in wild rice [3]. Auxin is another key plant hormone in rice panicle development. The *PAY1* gene improves secondary branching, grains per panicle, and grain yield per plant, by influencing the activity of polar auxin transport and altering distribution of indole-3-acetic acid [4]. In recent years more attention was concentrated on BR research in rice. *GNS4*, a novel allele of *DWARF11*, significantly enhances grain number and grain size by regulating the expression levels of BR synthesis and BR response related genes [5]. *MRG702* also regulates grains per panicle and grain shape by affecting BR biosynthesis and signal transduction [6]. *OsRLCK57* interacts with *OsBRI1* (a rice BR receptor) and negatively regulates BR signaling, which affects rice panicle secondary branching [7].

Among cloned rice panicle trait genes, some show pleiotropic effects on tiller or heading date and plant height. In some cases, tillers and panicle branches are consistently regulated by a single gene. *MONOCULM 1* (*MOC1*) is an important example in rice, the *moc1* mutant plants have fewer tillers and panicle branches [8]. Similarly, *LAX1* regulates both tillers and panicle branches in coordination [9]. However, in some cases, the number of tillers or panicle branches changes in the opposite way. For example, *IPA1* (*Ideal Plant Architecture 1*) encodes *OsSPL14* and is regulated by *OsmiR156*. *OsSPL14* mutation perturbs *OsmiR156* direct regulation of *OsSPL14*, which results in reduced tiller number and increased panicle branches [10]. This is similar to *PAY1*, where the *PAY1* mutant displays reduced tiller numbers and increased panicle branches [4]. These findings suggest that rice tillers and panicle branches may be regulated by distinct mechanisms. Some grain number regulation genes demonstrate pleiotropic roles in regulating heading date and plant height. *Ghd7* encodes a CCT domain protein, and has a significance effect on grain number. By regulating *Ehd1* and *Hd3a*, *Ghd7* affects heading date under long-day conditions. *Ghd7* can also enhance plant height by increasing cell numbers [11]. *Ghd8*/*DTH8* and *Ghd7*.*1*/*DTH7* also have pleiotropic effects on grain number, heading date, and plant height [12–15].

*OsGRF4*, an important pleiotropic gene can significantly enhance rice yield, and was recently cloned. *OsGRF4* encodes the growth-regulating factor 4, a transcription activator, which improve grain size, panicle length, and seed shattering. A rare nucleobase polymorphism in *OsGRF4* in the target site of *OsmiRNA396* is correlated to high expression of *OsGRF4*, which leads to the phenotypic changes in panicle traits [16–18]. *OsGSK2* negatively regulates transcription activation activity of *OsGRF4* by direct interaction; *OsGRF4* also interacts with GRF-interacting factor 1 (*OsGIF1*), which results in bigger grain [19]. The *OsmiR396c*-*OsGRF4*-*OsGIF1* regulatory pattern influences grain shape and improves rice yield [20]. Field studies show that *OsGRF4* can increase yield of hybrid rice (13.7%–28.0% increase) [16, 20]. Despite this, the molecular mechanisms of *OsGRF4* in regulating panicle traits remain elusive.

RNA sequencing technology (RNA-Seq) is a promising means for genome-wide transcriptomic analysis. Recently, RNA-Seq has been widely applied to transcriptional analyses associated with rice fertility, heterosis, drought tolerance, salinity stress, heat stress, cold tolerance and biotic stress [21–24]. In consideration of *OsGRF4* is a notable panicle trait gene and has broad application prospects in rice breeding. In this study, to illuminate gene expression regulatory networks involved in panicle trait development, we performed a comprehensive transcriptomic analysis between NIL-*OsGRF4* and NIL-*Osgrf4* in young panicles that were 7 cm long using RNA-seq. We detected over 331 million raw reads, of which roughly 317 million reads were clean. The error rate of all samples was very low (0.02%). In total, we identified 80 significant differentially expressed genes (DEGs). Hierarchical Cluster analysis showed that *OsGRF4* was tightly clustered with the up-regulated DEG LOC_Os02g47320, those two genes have highly similar gene expression pattern and LOC_Os02g47320 may play a crucial part in panicle trait regulation. We searched the reported functions of DEGs and compared them with previous identified quantitative trait loci (QTL) influencing panicle traits and stress tolerance. Out result suggest that the pleiotropic gene *OsGRF4* may be involved in abiotic resistance. The novel candidate genes identified here may play a significant role in panicle traits regulation, and our results provide valuable information for understanding the molecular mechanisms underlying rice panicle trait development.

## Materials and methods

### Plant materials

The big-grain rice variety CDL (contains *OsGRF4*) was crossed with the medium-grain variety R1126 (contains *Osgrf4*, an allele of *OsGRF4*) to obtain F_1_ plants, and F_10_ population of recombined inbred lines (RIL) were obtained by single-seed descent. Grain shape difference was observed in one F_10_ line (L28), and L28 showing heterozygous at *OsGRF4* locus was selfed to yield NIL-*OsGRF4* and NIL-*Osgrf4*.

### RNA isolation

Young panicles 7 cm in length were collected from NIL-*OsGRF4* and NIL-*Osgrf4* for RNA isolation and total RNA was extracted using TRlzol Reagent according to the manual instruction (Life technologies, California, USA). Each sample had three biological replicates. The concentration and purity of each RNA sample were assessed by gel electrophoresis and a NanoPhotometer^®^ spectrophotometer (IMPLEN, CA, USA). RNA integrity was assessed with an Agilent 2100 Bioanalyzer (Agilent, Palo Alto, CA, USA). RNA was used for RNA-seq and qRT-PCR.

### Tanscriptome sequencing

Sequence libraries were constructed by the Novogene Bioinformatics Institute (Beijing, China) and sequenced using the Illumina HiSeq^™^ 2000 platform. Low quality reads (unknown nucleotides and adaptor sequences) were filtered to get clean reads. Clean reads were mapped to the reference genome (ftp://ftp.ensemblgenomes.org/pub/release-20/plants/fasta/oryza_indica/dna/) using Tophat v2.0.9 software (Broad Institute, Cambridge, MA, USA).

### Differential expression analysis

The reads numbers mapped to each gene were counted using HTSeq v0.6.1 software, and gene expression levels were calculated using the reads per kb per million reads (RPKM) values. Differential gene expression analysis between NIL-*OsGRF4* and NIL-*Osgrf4* plants was performed by the DESeq R package, filtering DEGs with |log2 (Fold Change)| > 1 and corrected *p*-value (padj) < 0.05. Gene ontology (GO) enrichment analysis of DEGs was implemented by the GOseq R package. GO terms with corrected *p*-value < 0.05 were considered significantly enriched. KOBAS software was used to test the statistical enrichment of DEGs in the Kyoto Encyclopedia of Genes and Genomes (KEGG) pathways. RNA-seq data were deposited in the National Center for Biotechnology Information Sequence Read Archive under accession number SRP131560.

### RNA-seq validation by quantitative real-time PCR

To validate RNA-seq DEG data, quantitative real-time PCR (qRT-PCR) was conducted on 24 randomly selected genes (12 down regulated and 12 up-regulated DEGs). Total RNA of NIL-*OsGRF4* and NIL-*Osgrf4* was reverse transcribed using the TransScript All-in-One First-Strand cDNA Synthesis SuperMix for quantitative PCR (qPCR) kit (TransGen Biotech, Beijing, China). The relative expression was analyzed from cycle threshold values using the 2^−ΔΔCt^ method with the rice Ubiquitin 5 gene used as a reference, and all of the results had three biological replicates. The 24 DEGs and primer sequences are listed in S1 Table.

## Results

### The effect of *OsGRF4* on panicle traits phenotype of rice

Rice grains of NIL-*OsGRF4* were significant bigger and heavier compared to that of NIL-*Osgrf4*. This difference resulted in an increased storage capacity (Grain weight × Spikelet number per panicle × Tiller number) in NIL-*OsGRF4* plants by 30.37%. *OsGRF4* also positively regulated panicle length, but resulted in lower seed setting percentage. Interestingly, *OsGRF4* also significantly improve shattering degree [18]. The two NILs had similar germination rates just after harvest, but NIL-*OsGRF4* had very low germination after storing at room temperature for one year (S1 Fig). This result suggests that *OsGRF4* may negative regulate germination.

### Whole genome transcriptome profiles of NIL-*Osgrf4* and NIL-*OsGRF4*

The young panicle stage is critical for panicle traits determination. *OsGRF4* had the highest expression levels in young panicles measuring 7 cm long, and the relative expression was different between NIL-*Osgrf4* and NIL-*OsGRF4* [18]. Therefore, we sequenced the transcriptome profiles of 7 cm young panicle from the two NILs. We detected over 331 million raw reads, and obtained roughly 317 million clean reads, accounting for 95.83% of the raw reads. The error rate was low for all samples (0.02%). The average Q20 and Q30 were 96.35% and 91.02%, respectively. After aligning clean reads to the rice *indica* varieties 9311 reference genome, a total of 80.24–81.37% of reads were mapped, 78.39% of mapped reads were unique, and 2.07%–2.65% of reads were mapped to multiple loci (Table 1). Significant correlation was detected among transcriptome data across all biological replicates of both from NIL-*OsGRF4* and NIL-*Osgrf4* with a correlation coefficient greater than 0.97. The correlation analysis also showed that NIL-*OsGRF4* and NIL-*Osgrf4* had similar genetic background (S2 Fig).

**Table 1:** Summary of sequencing data generated and mapped to the reference genome.

Sample name	N_DLA	N_DLB	N_DLC	N_XLA	N_XLB	N_XLC
Raw reads	53581034	55079100	49357768	58703882	61334078	53044476
Clean reads	51478186	52937908	47535552	56109570	58756544	50471646
Clean bases	7.72G	7.94G	7.13G	8.42G	8.81G	7.57G
Error rate (%)	0.02	0.02	0.02	0.02	0.02	0.02
Q20 (%)	96.17	96.46	96.44	96.36	96.3	96.38
Q30 (%)	90.66	91.23	91.14	91.07	90.88	91.12
GC content (%)	51.85	51.94	51.87	52.56	51.32	52.24
Total mapped	41413972 (80.45%)	43060233 (81.34%)	38681326 (81.37%)	45023405 (80.24%)	47301997 (80.51%)	40517078 (80.28%)
Multiple mapped	1086373 (2.11%)	1346765 (2.54%)	995857 (2.09%)	1163241 (2.07%)	1418520 (2.41%)	1336574 (2.65%)
Uniquely mapped	40327599 (78.34%)	41713468 (78.8%)	37685469 (79.28%)	43860164 (78.17%)	45883477 (78.09%)	39180504 (77.63%)

N_DL: NIL-*OsGRF4*; N_XL: NIL-*Osgrf4*; A, B and C represents three biological replicates.

### Identification of DEGs between NILs

**Fig 1:**
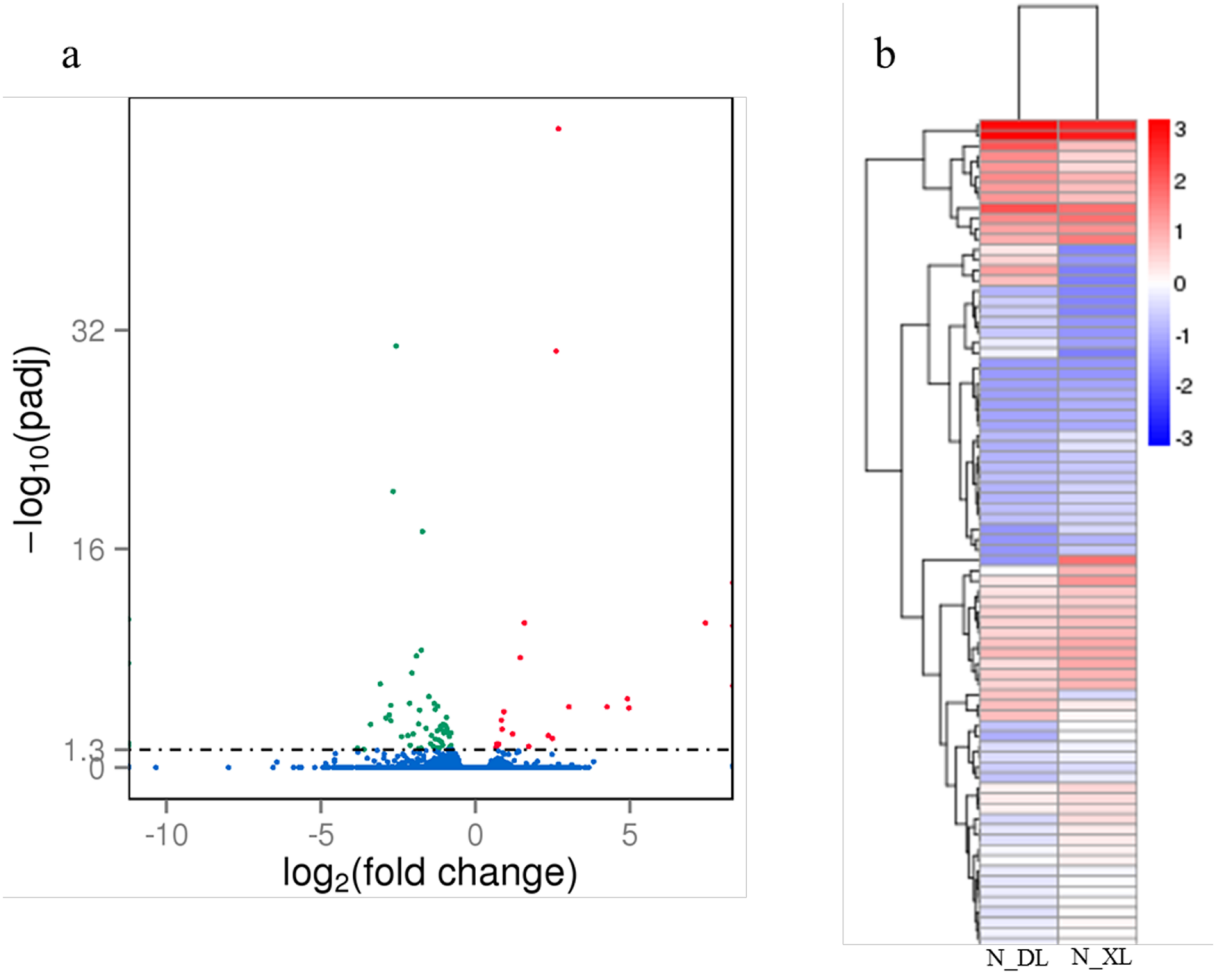
The overall distribution and Hierarchical clustering analysis of differentially expressed genes. **(a)** Red and green color represents 23 significant up-regulated DEGs and 57 significant down-regulated DEGs, respectively. **(b)** Red color shows high expression level genes and blue color shows low expression level genes. N_DL: NIL-*OsGRF4*; N_XL: NIL-*Osgrf4*.

We detected 24807 and 25370 expressed genes in NIL-*OsGRF4* and NIL-*Osgrf4*, respectively. A total of 24280 genes were common to both genotypes, with 527 and 1090 genes uniquely expressed in NIL-*OsGRF4* and NIL-*Osgrf4*, respectively (S3 Fig). In total, 80 significant DEGs were detected between NIL-*OsGRF4* and NIL-*Osgrf4*, including 23 up-regulated genes and 57 down-regulated genes (Fig 1a; S2 Table). RNA-seq showed the expression level of the target gene *OsGRF4* (LOC_Os02g47280 / BGIOSGA005785) was up-regulated in NIL-*OsGRF4* plant, which agrees with our previously reported qPCR analysis [18]. Hierarchical cluster analysis of DEGs showed that *OsGRF4* was tightly clustered with LOC_Os02g47320 (S4 Fig), showing that these two genes have similar gene expression patterns. NIL-*OsGRF4* and NIL-*Osgrf4* clustered together (Fig 1b), which revealed analogous genetic backgrounds.

### Expression patterns of DEGs were validated by qRT-PCR

**Fig 2:**
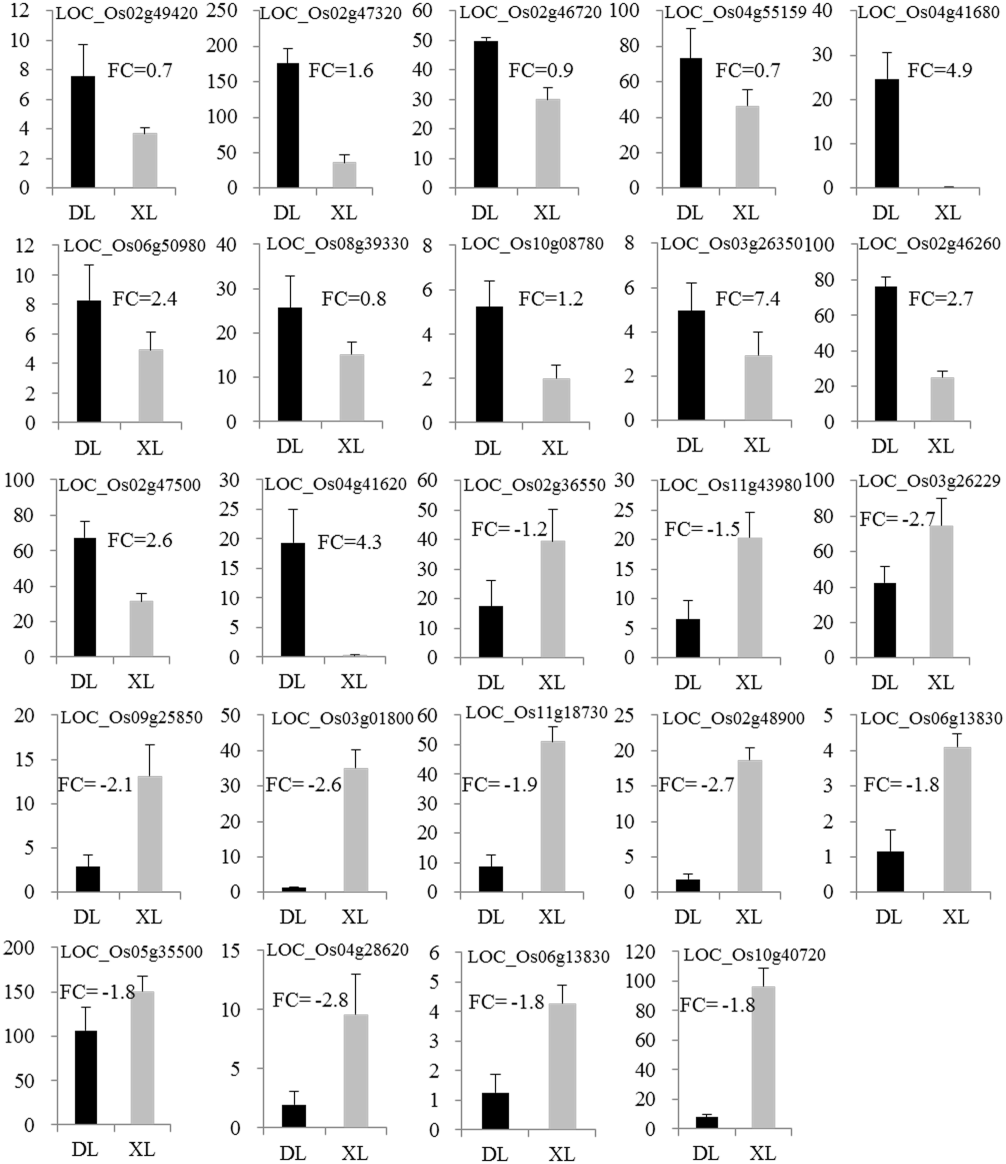
Expression patterns of DEGs were validated by qRT-PCR. The relative expressions of 24 DEGs (12 up-regulated genes and 12 down-regulated genes) were analyzed by real-time PCR with 7 cm young panicle. The rice Ubiquitin 5 gene (*OsUBQ5*) was used as a reference, results are showed as means ± SD with three biological replicates. FC: Fold change from transcriptome analysis. DL: NIL-*OsGRF4*; XL: NIL-*Osgrf4*.

To confirm DEGs identified in our transcriptome analysis, 24 randomly selected DEGs were analyzed by qRT-PCR. The qRT-PCR analysis showed that the 12 up-regulated DEGs (such as LOC_Os02g47320, LOC_Os04g55159 and LOC_Os04g41680, which involved in vacuolar ATPase G subunit, seed storage and chitinase family protein precursor) demonstrated higher expression levels in NIL-*OsGRF4* than in NIL-*Osgrf4* (Fig 2). The 12 down regulated DEGs (such as LOC_Os05g35500, LOC_Os04g28620 and LOC_Os09g25850, which involved in MYB transcription factor, male sterility and uncharacterised domain Wax2) showed lower expression levels in NIL-*OsGRF4* than in NIL-*Osgrf4*. Expression trends were consistent for all transcripts in qRT-PCR and RNA-Seq analyses.

### Functional classification of DEGs by GO analyzing

**Fig 3:**
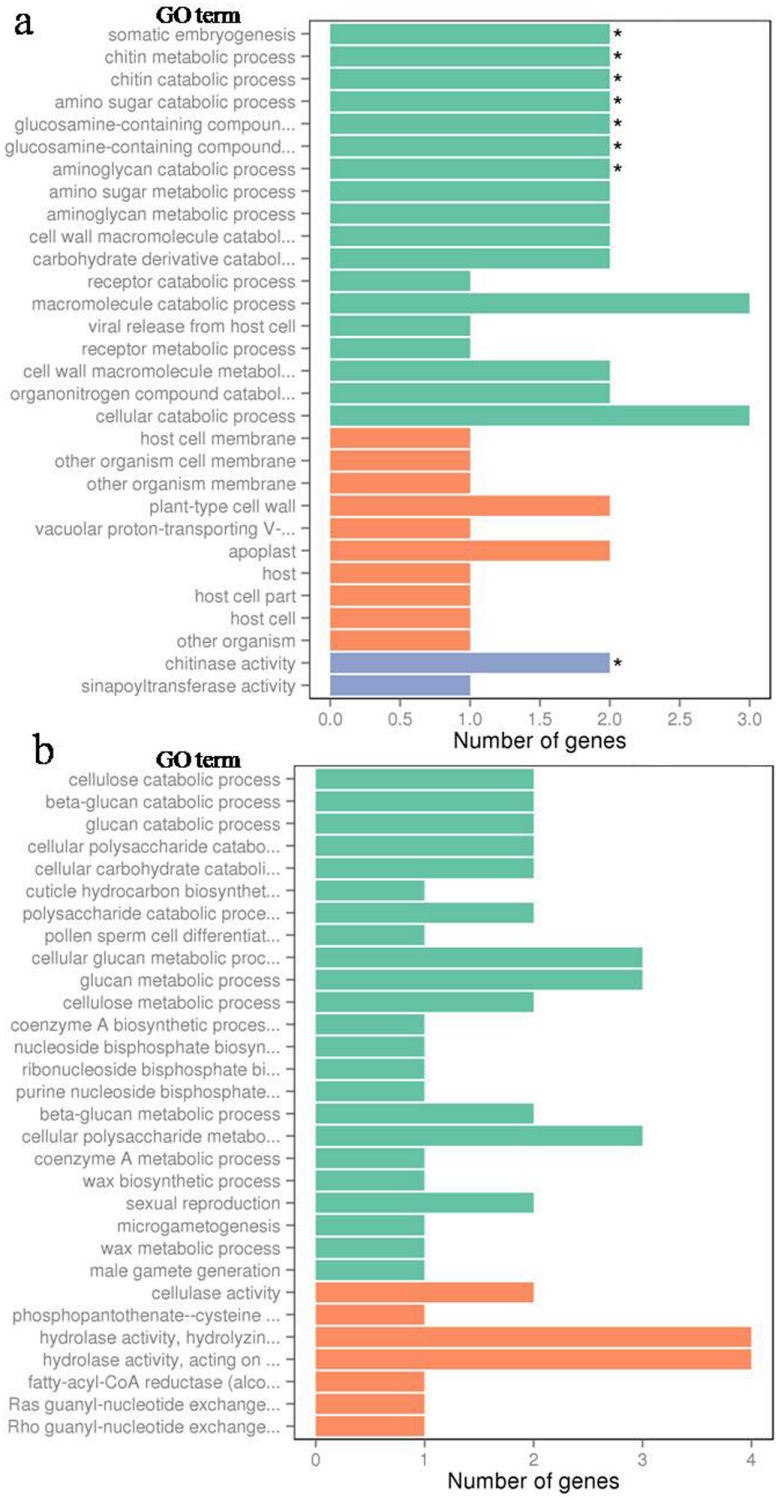
Gene ontology classifications of DEGs. (**a, b)** The most enriched GO categories in up-regulated DEGs and down-regulated DEGs, respectively. * GO terms were significantly enriched. Green, blue and red color represents biological process, molecular function and cellular component, respectively.

Gene ontology analysis of up-regulated genes showed 10 DEGs were enriched in 230 GO terms, including 63 functional terms in molecular function, 46 in cellular components, and 121 in biological processes. Our target gene *OsGRF4* was enriched in 83 functional terms, and over-represented (*P* < 0.05) in 2 GO terms, hydrolase activity (GO:0016787) and macromolecule metabolic process (GO:0043170) (S3 Table). Eight significantly enriched GO terms (corrected *P*-value < 0.05) were detected in up-regulated DEGs (Table 2, S3 Table, Fig 3a, S5 and S6 Figs), showing possible biological processes and molecular function underlying panicle trait regulation. Among the down-regulated genes, 36 DEGs were enriched in 296 GO terms. We further identified 47 over-represented GO terms, of which the 5 most highly enriched GO terms were cellulase activity (GO:0008810), cellulose catabolic process (GO:0030245), beta-glucan catabolic process (GO:0051275), glucan catabolic process (GO:0009251) and cellular polysaccharide catabolic process (GO:0044247) (Fig 3b, S4 Table).

**Table 2:** The significantly enriched GO terms in the RNA-seq analysis.

Term	GO_accession	Sample number	Background number	Corrected *P*-Value
Somatic embryogenesis	GO:0010262	2	10	0.011
Chitinase activity	GO:0004568	2	10	0.039
Chitin metabolic process	GO:0006030	2	10	0.039
Chitin catabolic process biosynthesis	GO:0006032	2	10	0.039
Amino sugar catabolic process	GO:0046348	2	10	0.039
Glucosamine-containing compound Metabolic process	GO:1901071	2	10	0.039
Glucosamine-containing compound Catabolic process	GO:1901072	2	10	0.039
Aminoglycan catabolic process	GO:0006026	2	10	0.041

### KEGG pathway analysis of DEGs

KEGG analysis showed 10 out of 80 DEGs were enriched across 17 functional categories. Among the up-regulated genes, one showed notable enrichment (corrected *P*-value < 0.05) in the pathways for amino sugar and nucleotide sugar metabolism (osa00520), suggesting this pathway may perform an important function in rice panicle development (S5 Table). Among the down-regulated genes, three over-represented KEGG terms (*P* < 0.05) were identified, including cutin, suberine and wax biosynthesis (osa00073), pantothenate and CoA biosynthesis (osa00770), and beta-Alanine metabolism (osa00410) (S6 Table). Subsequent functional analysis of DEGs involved in the KEGG pathway may help elucidate the complex regulatory networks of panicle traits in rice.

### Chromosome co-localization of DEGs with previously identified QTL responsible for panicle traits

**Fig 4:**
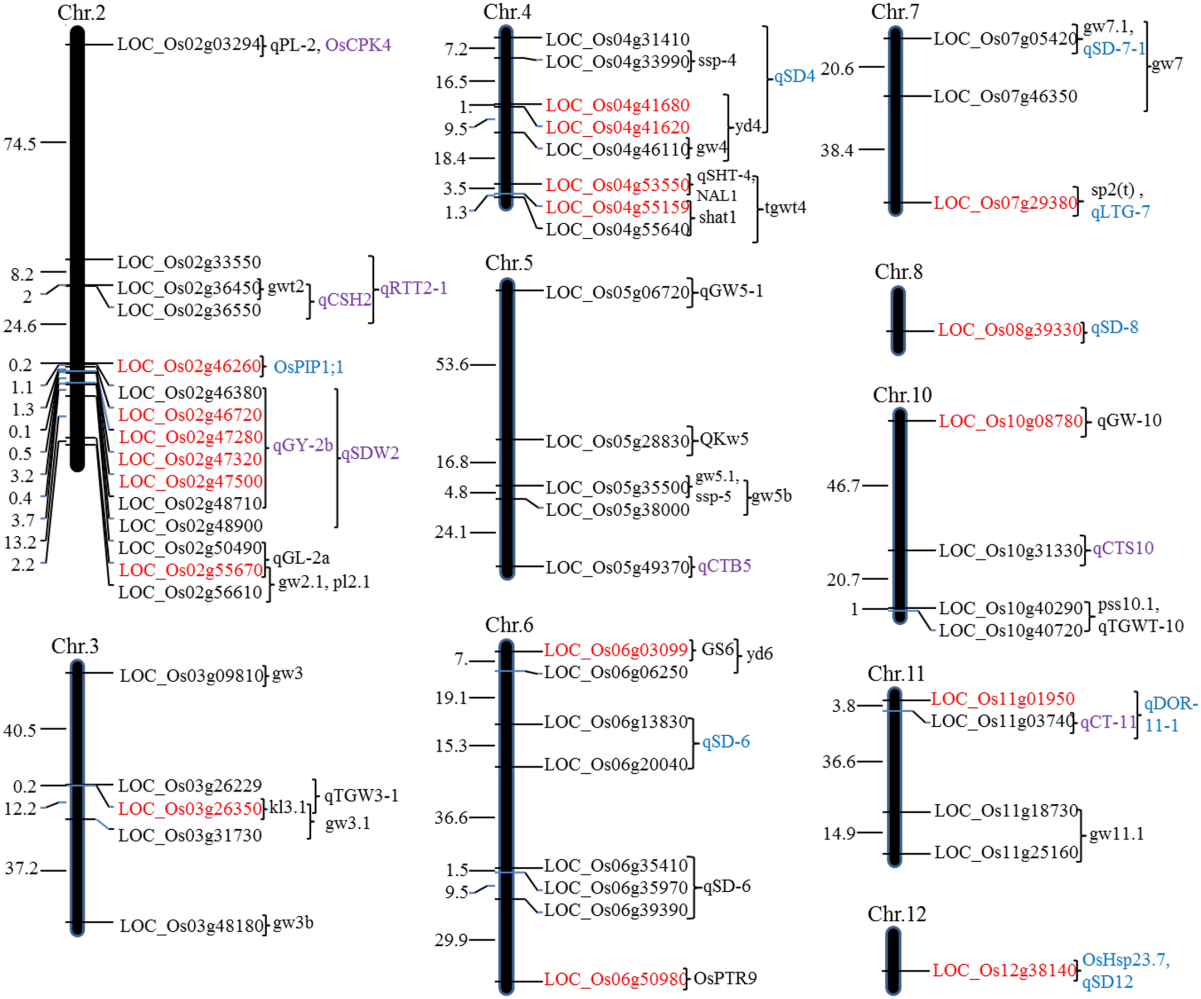
The DEGs co-localized with previous identified panicle traits and tolerance QTLs onto rice chromosomes. For the DEGs, red color represents up-regulated genes, black color represents down-regulated genes; For the QTLs, genes in black, blue and purple letters associated with panicle traits, seed germination/dormancy and cold/drought tolerance, respectively.

To identify the potential function of DEGs, we co-localized previously reported QTLs (http://qtaro.abr.affrc.go.jp/, http://archive.gramene.org/) and DEGs onto rice chromosomes. A total of 54 DEGs (18 up-regulated and 36 down-regulated) were co-localized within 50 QTLs intervals. Among them, 22 QTLs were responsible for grain size or grain weight, 4 for panicle length, 2 for seed shattering, 2 for seed set percent, 9 for seed germination/dormancy, and 11 for cold/drought tolerance. Chromosome 2 had the greatest number of co-localized QTLs with 11 QTLs, followed by chromosome 4 with 8 QTLs (Fig 4, S7 Table).

Three up-regulated DEGs (LOC_Os02g55670, LOC_Os03g26350, and LOC_Os04g53550) co-localized with 3 panicle traits QTLs, respectively. The pleiotropic QTLs *SLCHL4* (*NAL1*) and *OsPTR9* controlling grain weight and panicle size [25, 26] were co-localized with LOC_Os04g53550 and LOC_Os06g50980, respectively. The up-regulated DEG LOC_Os04g41680 belonged to the significantly enriched KEGG pathway (S5 Table) and co-localized with *yd4* responsible for grain yield [27], which suggests that LOC_Os04g41680 may be a candidate panicle trait QTL, playing an important role in the regulatory network of panicle traits by differential expression levels. We also found some DEGs were located in cold/drought tolerance-related QTL regions (Fig 4, S7 Table). For instance, pleiotropic QTLs *OsHsp23*.*7* and *qLTG-7* were not only responsible for seed germination/dormancy but also for cold/drought and salt tolerance [28, 29]. Interestingly, our target gene *OsGRF4* (LOC_Os02g47280) was located in a cold tolerance QTL *qSDW2* [30] and a drought tolerance QTL *qGY-2b* [31]. This suggests that the pleiotropic gene *OsGRF4* may be involved in abiotic stress resistance. A previous study showed that *OslecRK* was responsible for both plant innate immunity and seed germination, and knocking out *OslecRK* reduced seed germination and resistance to bacterial and fungal pathogens as well as insects in rice [32]. We found a down-regulated gene (LOC_Os05g35500) encoding the Myb transcription factor *Osmyb1*, which may be involved in trans-regulation, was co-localized with 3 QTLs responsible for grain weight (QTL *gw5b* and *gw5*.*1*) and seed set percent (QTL *ssp-5*) [33–35]. In summary, the co-localized DEGs identified in this study provide a starting point for elucidating the molecular regulation mechanisms of rice panicle traits.

## Discussion

Panicle traits such as panicle shape, grain size, seed germination/dormancy, and seed shattering are important to rice yield and quality. Although a number of genes controlling panicle traits have been isolated, the genetic regulation mechanisms underlying them are still uncertain. Gene expression profiling analysis is a good method for understanding the molecular mechanisms involved in panicle traits regulation. At the same time, current sequencing technologies provide high throughput, accurate, and economical methods to study whole transcriptomes. In recent years, RNA-seq has been widely applied to investigate the transcriptome of numerous plants species across a range of environment conditions, including rice [22], maize [36], sorghum [37], tea plant [38], bamboo [39] and wild species as well [40, 41]. NILs are ideal material for detecting DEGs, having a single gene difference between two genotypes, which eliminates most genetic background noise. For example, Raorane et al analyzed the proteome of spikelets, roots, and flag leaves between NILs with different drought resistant phenotypes and identified DEGs under drought conditions [42]. Similarly, we performed a transcriptomics sequence analysis of young rice panicles between NIL-*Osgrf4* and NIL-*OsGRF4* to study molecular mechanisms of panicle trait regulation. By comprehensively analysis of RNA-seq data, we provide new insights into panicle trait regulation in rice.

Our RNA-Seq analysis identified 80 significant DEGs, including 23 up-regulated DEGs and 57 down-regulated DEGs. The target gene *OsGRF4* (LOC_Os02g47280) was up-regulated according to RNA-Seq results, which is consistent with our previous qPCR results [18]. To evaluate the potential role of identified DEGs, we searched reported functions in existing databases. Eighteen DEGs were reported to be involved in panicle trait regulation in rice. The down-regulated DEG *BSG1*/*TH1* (LOC_Os02g56610) encodes a DUF640 domain protein and determines grain width, thickness, and yield by regulating expression levels of cell expansion and division genes [43, 44]. *BSG1*/*TH1* also co-localized with 100-grain weight QTL *gw2*.*1* and panicle length QTL *pl2*.*1* [45]. Rice grain production is also regulated by cytokinin content [2], and we identified four cytokinins significant differences between NIL-*OsGRF4* and NIL-*Osgrf4* [18]. The purine permease family (*OsPUP*) is involved in cytokinin transporter and has 12 members in rice. *OsPUP7* mutants had high cytokinin content, which resulted in larger seeds and increasing sensitivity to salt and drought stresses [46]. The down-regulated DEG *OsPUP11* (LOC_Os02g46380), is also a cytokinin transporter and may influence grain shape. Polyamine is important for normal seed development in Arabidopsis [47] and polyamine oxidase (PAO) is a FAD-dependent enzyme associated with polyamine catabolism. The rice genome has 7 PAOs [48], and of these, the down-regulated DEG *OsPAO2* (LOC_Os03g09810) co-localized with seed weight QTL *gw3* [49], thus, *OsPAO2* may influence grain weight. DEG LOC_Os02g50490 was mainly detected during seed and panicle developmental (stages −5, −6), and therefore may be involved in panicle trait regulation [50].

Transcription factors (TFs) are regulatory proteins and plays a crucial role in panicle trait development [51, 52]. The seed shattering gene *sh4* encodes a nuclear TF that is a member of trihelix TF family, which may be derived from the MYB gene family [53]. Four up-regulated DEGs: LOC_Os02g49420, LOC_Os04g41680, LOC_Os04g41620, and LOC_Os08g39330, are up-regulated by *sh4* [54]. In plants, MYB TFs have been isolated from many species, and serve various functions including involvement in development, primary and secondary metabolism, flowering time, and stresses response [55, 56]. The *Ant28* gene encodes a R2R3 MYB protein which influences barley seed dormancy [57]. *MYB56* encodes a R2R3 MYB TF, which positively controls seed size in *A*. *thaliana* in an unknown pathway [58]. *Osmyb1* (LOC_Os05g35500) was down-regulated in this study and was found to be co-localized with 3 QTLs which determine grain weight (QTL *gw5b* and *gw5*.*1*) and seed set percent (and QTL *ssp-5*) [33–35]. Phylogeny analysis showed that *AtMYB66* (encoding the R2R3 MYB protein) had the closest genetic relationship with *Osmyb1* [59]. The expression levels of *Osmyb1* and *Osmyb4* peaked in seeds at 14 days after flowering, which suggests that these two genes may function in seed maturation [60]. Other studies have shown that *Osmyb1* is associated with cold and drought tolerance [61, 62]. Therefore, the *Osmyb1* TF may act as a key regulator of panicle traits and abiotic resistance.

The pleiotropic gene *OsGRF4* may also affect other phenotype besides panicle trait. It was reported that *OsGRF4* (LOC_Os02g47280) was a target gene of *osa-MIR396c*, and overexpression of *osa-MIR396c* was shown to reduce alkali and salt stress tolerance. *OsGRF4* was up-regulated when treated by saline, anoxia, ABA, and gibberellins, and down-regulated by arsenate [63]. Another study found that LOC_Os02g47280 showed differential expression in root knot nematode infection [64]. Interestingly, we found that *OsGRF4* co-localized with both a cold tolerance QTL (*qSDW2*) [30] and a drought tolerance QTL (*qGY-2b*) [31]. Many DEGs detected in this study, such as LOC_Os04g55159, LOC_Os04g41680, LOC_Os04g41620, LOC_Os04g53550, *OsGL1-1* (LOC_Os09g25850), LOC_Os10g31330 and LOC_Os04g33990, were found to be regulated by biotic and abiotic stress such as cold, salt, and drought stress as well as blast disease infection [65–69]. The *Osmyb1* TF not only influences seed maturation [60], but was also found to be associated with cold and drought tolerance [61, 62]. Therefore, *OsGRF4* and some DEGs detected in this study may be involved in abiotic and biotic stress responses, which need further investigation.

In our GO enrichment analysis, two up-regulated DEGs (LOC_Os04g41680 and LOC_Os04g41620) were significantly enriched in the top 8 GO terms, and were Over_represented in term of seed development (GO: 0048316) (S3 Table). Therefore, LOC_Os04g41680 and LOC_Os04g41620 may be important genes regulating grain shape. Along with LOC_Os04g41680 and LOC_Os04g41620, three up-regulated genes (*OsGRF4*, LOC_Os02g47320, and LOC_Os02g46260) and 9 down-regulated genes (LOC_Os02g50490, LOC_Os03g01800, LOC_Os02g48900, LOC_Os12g04320, LOC_Os10g05910, LOC_Os06g13830, LOC_Os06g06250, LOC_Os05g06720, and LOC_Os07g46350) were enriched for hydrolase activity (GO: 0016787) (S3 and S4 Tables). *TGW6* encodes a novel protein that has indole-3-acetic acid-glucose hydrolase activity, and enhances rice seed weight and yield [70]. Therefore, DEGs enriched in hydrolase activity GO term may be potential candidate genes for grain shape. *OsGRF4* and LOC_Os02g47320 were collectively enriched in 14 GO terms (S3 Table), and LOC_Os02g47320 was also involved in 3 KEGG pathways (S5 Table). In addition, *OsGRF4* was tightly clustered with LOC_Os02g47320 (S4 Fig), which shows that these two genes have highly similar gene expression pattern and LOC_Os02g47320 may play a crucial role in panicle trait regulation, however further research is needed.

In summary, our transcriptome sequence analyses provide some important candidate genes involving in panicle trait regulation, which may be used in future gene cloning studies. Functional analysis of the DEGs and pathways identified here will serve to further our understanding of the transcriptional network and molecular mechanisms underlying panicle trait regulation in rice.

## Supporting information

S1 Fig Seed germination percentage of NIL-*OsGRF4* and NIL-*Osgrf4*. The seeds have been stored at room temperature over one year.
(TIF)

S2 Fig Pearson correlation coefficient between samples. N_DL: NIL-*OsGRF4*; N_XL: NIL-*Osgrf4*; A, B and C represents three biological replicates.
(TIF)

S3 Fig Venn diagram showing the genes expressed in NIL-*OsGRF4* and NIL-*Osgrf4*. A total of 24807 and 25370 genes with expression were identified from NIL-*OsGRF4* and NIL-*Osgrf4*, respectively. N_DL: NIL-*OsGRF4*; N_XL: NIL-*Osgrf4*.
(TIF)

S4 Fig Cluster analysis of differentially expressed genes. *OsGRF4* (BGIOSGA005785) tightly clustered together with LOC_Os02g47320 (BGIOSGA005784). N_DL: NIL-*OsGRF4*; N_XL: NIL-*Osgrf4*.
(TIF)

S5 Fig GO analysis of differentially expressed genes of the biological process category. (TIF)

S6 Fig GO analysis of differentially expressed genes of the molecular function category. (TIF)

S1 Table qRT-PCR primer sequence. (XLSX)

S2 Table A total of 80 differentially expressed genes identified between NIL-*OsGRF4* and NIL-*Osgrf4*. (XLSX)

S3 Table GO enrichment analysis for the up-regulated genes. (XLSX)

S4 Table GO enrichment analysis for the down-regulated genes. (XLSX)

S5 Table NIL-*OsGRF4* vs NIL-*Osgrf4* up-regulated DEGs KEGG_pathway. (XLSX)

S6 Table NIL-*OsGRF4* vs NIL-*Osgrf4* down-regulated DEGs KEGG_pathway. (XLSX)

S7 Table List of previously identified panicle traits and tolerance QTLs downloaded from QTARO and gramene database. (XLSX)

## References

[1] Khush GS. What it will take to feed 5.0 billion rice consumers in 2030. Plant Mol Biol. 2005; 59: 1–6. doi: 10.1007/s11103-005-2159-5 16217597 10.1007/s11103-005-2159-5

[2] Ashikari M, Sakakibara H, Lin SY, Toshio Y, Tomonori T, Asuka N, et al Cytokinin oxidase regulates rice Grain production. Science. 2005; 309 (741): 741–745. 15976269 10.1126/science.1113373

[3] Jin J, Hua L, Zhu ZF, Tan LB, Zhao XH, Zhang WF, et al *GAD1* Encodes a Secreted Peptide That Regulates Grain Number, Grain Length, and Awn Development in Rice Domestication. The Plant Cell. 2016; 28: 2453–2463. doi: 10.1105/tpc.16.00379 27634315 10.1105/tpc.16.00379 PMC5134979

[4] Zhao L, Tan LB, Zhu ZF, Xiao LT, Xie DX, Sun CQ. *PAY1* improves plant architecture and enhances grain yield in rice. The Plant J. 2015; 83(3): 528–536. doi: 10.1111/tpj.12905 26095647 10.1111/tpj.12905 PMC4758413

[5] Zhou Y, Tao YJ, Zhu JY, Miao J, Liu J, Liu YH, et al *GNS4*, a novel allele of DWARF11, regulates grain number and grain size in a high-yield rice variety. Rice. 2017; 10:34 doi: 10.1186/s12284-017-0171-4 28730412 10.1186/s12284-017-0171-4 PMC5519514

[6] Jin J, Shi JL, Liu B, Liu YC, Huang Y, Yu Y, et al MORF-RELATED GENE702, a Reader Protein of Trimethylated Histone H3 Lysine 4 and Histone H3 Lysine 36, Is Involved in Brassinosteroid-Regulated Growth and Flowering Time Control in Rice. Plant Physiol. 2015; 168(4): 1275–1285. doi: 10.1104/pp.114.255737 25855537 10.1104/pp.114.255737 PMC4528726

[7] Zhou XG, Wang J, Peng CF, Zhu XB, Yin JJ, Li WT, et al Four receptor-like cytoplasmic kinases regulate development and immunity in rice. Plant, Cell & Environment. 2016; 39(6): 1381–1392. 10.1111/pce.12696 26679011

[8] Li XY, Qian Q, Fu ZM, Wang YH, Xiong GS, Zeng DL, et al Control of tillering in rice. Nature. 2003; 422 (6932): 618–621. doi: 10.1038/nature01518 12687001 10.1038/nature01518

[9] Keishi K, Masahiko M, Shin U, Yuzuki S, Ikuyo F, Hironobu O, et al *LAX* and *SPA*: Major regulators of shoot branching in rice. Proc Natl Acad Sci USA. 2003; 100 (20): 11765–11770. doi: 10.1073/pnas.1932414100 13130077 10.1073/pnas.1932414100 PMC208832

[10] Jiao YQ, Wang YH, Xue DW, Wang J, Yan MX, Liu GF, et al Regulation of *OsSPL14* by *OsmiR156* defines ideal plant architecture in rice. Nature Genet. 2010; 42(6): 541–544. doi: 10.1038/ng.591 20495565 10.1038/ng.591

[11] Xue WY, Xing YZ, Weng XY, Zhao Y, Tang WJ, Wang L, et al Natural variation in *Ghd7* is an important regulator of heading date and yield potential in rice. Nature Genet. 2008; 40, 761–767. doi: 10.1038/ng.143 18454147 10.1038/ng.143

[12] Yan WH, Wang P, Chen HX, Zhou HJ, Li QP, Wang CR, et al A major QTL, *Ghd8*, plays pleiotropic roles in regulating grain productivity, plant height, and heading date in rice. Mol Plant. 2011; 4, 319–330. doi: 10.1093/mp/ssq070 21148627 10.1093/mp/ssq070

[13] Wei XJ, Xu JF, Guo HN, Jiang L, Chen SH, Yu CY, et al *DTH8* suppresses flowering in rice, influencing plant height and yield potential simultaneously. Plant Physiol. 2010; 153: 1747–1758. doi: 10.1104/pp.110.156943 20566706 10.1104/pp.110.156943 PMC2923886

[14] Yan WH, Liu HY, Zhou XC, Li QP, Zhang J, Lu L, et al Natural variation in *Ghd7*.*1* plays an important role in grain yield and adaptation in rice. Cell Res. 2013; 23 (7): 969–971. doi: 10.1038/cr.2013.43 23507971 10.1038/cr.2013.43 PMC3698629

[15] Gao H, Jin MN, Zheng XM, Chen J, Yuan DY, Xin YY, et al *Days to heading 7*, a major quantitative locus determining photoperiod sensitivity and regional adaptation in rice. Proc Natl Acad Sci USA. 2014; 111 (46): 16337–16342. doi: 10.1073/pnas.1418204111 25378698 10.1073/pnas.1418204111 PMC4246261

[16] Hu J, Wang YX, Fang YX, Zeng LJ, Xu J, Yu HP, et al A rare allele of *GS2* enhances grain size and grain yield in rice. Mol Plant. 2015; 8: 1455–1465. doi: 10.1016/j.molp.2015.07.002 26187814 10.1016/j.molp.2015.07.002

[17] Duan PG, Ni S, Wang JM, Zhang BL, Xu R, Wang YX, et al Regulation of *OsGRF4* by *OsmiR396* controls grain size and yield in rice. Nat Plants. 2015; doi: 10.1038/NPLANTS.2015.203 27250749 10.1038/nplants.2015.203

[18] Sun PY, Zhang WH, Wang YH, He Q, Shu F, Liu H, et al *OsGRF4* controls grain shape, panicle length and seed shattering in rice. J Integr Plant Biol. 2016; 58: 836–847. doi: 10.1111/jipb.12473 26936408 10.1111/jipb.12473 PMC5089622

[19] Che RH, Tong HN, Shi BH, Liu YQ, Fang SR, Liu DP, et al Control of grain size and rice yield by *GL2*-mediated brassinosteroid responses. Nat Plants. 2015; doi: 10.1038/NPLANTS.2015.195 27250747 10.1038/nplants.2015.195

[20] Li SC, Gao FY, Xie KL, Zeng XH, Cao Y, Zeng J, et al The *OsmiR396c*-*OsGRF4*-*OsGIF1* regulatory module determines grain size and yield in rice. Plant Biotechnology J. 2016; doi: 10.1111/pbi.12569 27107174 10.1111/pbi.12569 PMC5095787

[21] Guo HB, Mendrikahy JN, Xie L, Deng JF, Lu ZJ, Wu JW, et al Transcriptome analysis of neo-tetraploid rice reveals specific differential gene expressions associated with fertility and heterosis. Scientific Reports. 2017; doi: 10.1038/srep40139 28071676 10.1038/srep40139 PMC5223177

[22] Shankar R, Bhattacharjee A, Jain M. Transcriptome analysis in different rice cultivars provides novel insights into desiccation and salinity stress responses. Scientific Reports. 2016; 6: 23719 doi: 10.1038/srep23719 27029818 10.1038/srep23719 PMC4814823

[23] Gonza N, Dreni L, Lawas L, Galbiati M, Colombo L, Heuer S, et al Genome-Wide Transcriptome Analysis During Anthesis Reveals New Insights into the Molecular Basis of Heat Stress. Plant Cell Physiol. 2016; 57: 57–68. doi: 10.1093/pcp/pcv174 26561535 10.1093/pcp/pcv174

[24] Dametto A, Sperotto R, Adamski J, Blasi É, Cargnelutti D, Oliveira L, et al Cold tolerance in rice germinating seeds revealed by deep RNAseq analysis of contrasting indica genotypes. Plant Sci. 2015; 238: 1–12. doi: 10.1016/j.plantsci.2015.05.009 26259169 10.1016/j.plantsci.2015.05.009

[25] Fang ZM, Xia KF, Yang X, Grotemeyer MS, Meier S, Rentsch D, et al Altered expression of the *PTR*/*NRT1* homologue *OsPTR9* affects nitrogen utilization efficiency, growth and grain yield in rice. Plant Biotechnology J. 2013; 11: 446–458. 10.1111/pbi.12031 23231455

[26] Zhang GH, Li SY, Wang L, Ye WJ, Zeng DL, Rao YC, et al *LSCHL4* from Japonica Cultivar, Which Is Allelic to *NAL1*, Increases Yield of Indica Super Rice 93–11. Mol Plant. 2014; 7: 1350–1364. doi: 10.1093/mp/ssu055 24795339 10.1093/mp/ssu055 PMC4115278

[27] Yu SB, Li JX, Xu CG, Tan YF, Gao YJ, Li XH, et al Importance of epistasis as the genetic basis of heterosis in an elite rice hybrid, Proc Natl Acad Sci USA. 1997; 94: 9226–9231. 11038567 10.1073/pnas.94.17.9226 PMC23127

[28] Zou J, Liu CF, Liu AL, Zou D, Chen XB. Overexpression of *OsHsp17*.*0* and *OsHsp23*.*7* enhances drought and salt tolerance in rice. J Plant Physiol. 2012; 169: 628–635. doi: 10.1016/j.jplph.2011.12.014 22321692 10.1016/j.jplph.2011.12.014

[29] Hou MY, Jiang L, Wang CM, Wan JM. Detection and analysis of QTLs for low temperature germinability in rice (Oryza sativa L.). Rice Genet Newsletter. 2003; 20: 52–55.

[30] Han L, Qiao Y, Zhang S, Zhang Y, Cao G, Kim J, et al Identification of quantitative trait loci for cold response of seedling vigor traits in rice. J Genet Genomics. 2007; 34: 239–246. doi: 10.1016/S1673-8527(07)60025-3 17498621 10.1016/S1673-8527(07)60025-3

[31] Zou GH, Mei HW, Liu HY, Liu GL, Hu SP, Yu XQ, et al Grain yield responses to moisture regimes in a rice population: association among traits and genetic markers. Theor Appl Genet. 2005; 112: 106–113. doi: 10.1007/s00122-005-0111-3 16231161 10.1007/s00122-005-0111-3

[32] Cheng XY, Wu Y, Guo JP, Du B, Chen RZ, Zhu LL, et al A rice lectin receptor-like kinase that is involved in innate immune responses also contributes to seed germination. The Plant J. 2013; 76: 687–698. doi: 10.1111/tpj.12328 24033867 10.1111/tpj.12328 PMC4285754

[33] Lu CF, Shen LH, Tan ZB, Xu YB, He P, Chen Y, et al Comparative mapping of QTLs for agronomic traits of rice across environments by using a doubled-haploid population. Theor Appl Genet. 1997; 94: 145–150. doi: 10.1007/s001220050393 19352757 10.1007/s001220050393

[34] Hua JP, Xing YZ, Xu CG, Sun XL, Yu SB, Zhang Q. Genetic dissection of an elite rice hybrid revealed that heterozygotes are not always advantageous for performance. Genetics. 2002; 162: 1885–1895. 12524357 10.1093/genetics/162.4.1885 PMC1462368

[35] Thomson MJ, Tai TH, McClung AM, Lai XH, Hinga ME, Lobos KB, et al Mapping quantitative trait loci for yield, yield components and morphological traits in an advanced backcross population between *Oryza rufipogon* and the *Oryza sativa* cultivar Jefferson. Theor Appl Genet. 2003; 107: 479–493. doi: 10.1007/s00122-003-1270-8 12736777 10.1007/s00122-003-1270-8

[36] Xu J, Yuan YB, Xu YB, Zhang GY, Guo XS, Wu FK, et al Identification of candidate genes for drought tolerance by whole- genome resequencing in maize. BMC Plant Biol. 2014; 14: 83 doi: 10.1186/1471-2229-14-83 24684805 10.1186/1471-2229-14-83 PMC4021222

[37] Fracasso A, Trindade LM, Amaducci S. Drought stress tolerance strategies revealed by RNA-Seq in two sorghum genotypes with contrasting WUE. BMC Plant Biol. 2016; 16: 115 doi: 10.1186/s12870-016-0800-x 27208977 10.1186/s12870-016-0800-x PMC4875703

[38] Li Y, Huang J, Song XW, Zhang ZW, Jiang Y, Zhu YL, et al An RNA-Seq transcriptome analysis revealing novel insights into aluminum tolerance and accumulation in tea plant. Planta. 2017; 246: 91–103. doi: 10.1007/s00425-017-2688-6 28365842 10.1007/s00425-017-2688-6

[39] Cui K, Wang HY, Liao SX, Tang Q, Li L, Cui YZ, et al Transcriptome Sequencing and Analysis for Culm Elongation of the World’s Largest Bamboo (*Dendrocalamus sinicus*). Planta. 2016; doi: 10.1371/journal.pone.0157362 27304219 10.1371/journal.pone.0157362 PMC4909198

[40] Jhanwar S, Priya P, Garg R, Parida SK, Tyagi AK, Jain M. Transcriptome sequencing of wild chickpea as a rich resource for marker development. Plant Biotechnol J. 2012; 10: 690–702. doi: 10.1111/j.1467-7652.2012.00712.x 22672127 10.1111/j.1467-7652.2012.00712.x

[41] Zhang FT, Zhou Y, Zhang M, Luo XD, Xie JK. Effects of drought stress on global gene expression profile in leaf and root samples of Dongxiang wild rice (*Oryza rufipogon*). Bioscience Reports. 2017); doi: 10.1042/BSR20160509 28424372 10.1042/BSR20160509 PMC6434088

[42] Raorane ML, Pabuayon IM, Varadarajan AR, Mutte SK, Kumar A, Treumann A, et al Proteomic insights into the role of the large-effect QTL*qDTY12*.*1* for rice yield under drought. Mol Breeding. 2015; 35:139.

[43] Yan DW, Zhou Y, Ye SH, Zeng LJ, Zhang XM, He ZH. BEAK-SHAPED GRAIN 1/TRIANGULAR HULL 1, a *DUF640* gene, is associated with grain shape, size and weight in rice. Science China Life Sciences. 2013; 56: 275–283. doi: 10.1007/s11427-013-4449-5 23526395 10.1007/s11427-013-4449-5

[44] Ren DY, Rao YC, Wu LW, Xu QK, Li ZZ, Yu HP, et al The pleiotropic *ABNORMAL FLOWER AND DWARF1* affects plant height, floral development and grain yield in rice. Journal of Integrative Plant Bio. 2016; 58: 529–539. 10.1111/jipb.12441 PMC5064741 26486996

[45] Marri PR, Sarla N, Reddy LV, Siddiq EA. Identification and mapping of yield and yield related QTLs from an Indian accession of *Oryza rufipogon*. BMC Genet. 2005; 6: 33 doi: 10.1186/1471-2156-6-33 15949048 10.1186/1471-2156-6-33 PMC1181812

[46] Qi ZY, Xiong LZ. Characterization of a Purine Permease Family Gene *OsPUP7* Involved in Growth and Development Control in Rice. J Integra Plant Bio. 2013; 55: 1119–1135. 10.1111/jipb.12101 24034337

[47] Urano K, Hoboa T, Shinozaki K. Arabidopsis ADC genes involved in polyamine biosynthesis are essential for seed development. FEBS Letters. 2005; 579 1557–1564. doi: 10.1016/j.febslet.2005.01.048 15733873 10.1016/j.febslet.2005.01.048

[48] Ono Y, Kim DW, Watanabe K, Sasaki A, Niitsu M, Berberich T, et al Constitutively and highly expressed *Oryza sativa* polyamine oxidases localize in peroxisomes and catalyze polyamine back conversion. Amino Acids. 2012; 42: 867–876. doi: 10.1007/s00726-011-1002-3 21796433 10.1007/s00726-011-1002-3

[49] Xiao JH, Li JM, Yuan LP, Tanksley SR. Identification of QTLs affecting traits of agronomic importance in a recombinant inbred population derived from a subspecific rice cross. THEOR APPL GENET. 1996; 92: 230–244. doi: 10.1007/BF00223380 24166172 10.1007/BF00223380

[50] Kundu S, Sharma R, Kundu S, Sharma R. In silico identification and taxonomic distribution of plant class C GH9 endoglucanases. Front Plant Sci. 2016; 7: 1185 doi: 10.3389/fpls.2016.01185 27570528 10.3389/fpls.2016.01185 PMC4981690

[51] Huang RY, Jiang LR, Zheng JS, Wang TS, Wang HC, Huang YM, et al Genetic bases of rice grain shape: so many genes, so little known. Trends Plant Sci. 2013; 18: 218–226. doi: 10.1016/j.tplants.2012.11.001 23218902 10.1016/j.tplants.2012.11.001

[52] Miura K, Ashikari M, Matsuoka M. The role of QTLs in the breeding of high-yielding rice. Trends Plant Sci. 2011; 16: 319–326. doi: 10.1016/j.tplants.2011.02.009 21429786 10.1016/j.tplants.2011.02.009

[53] Nagano Y. Several features of the GT-factor trihelix domain resemble those of the Myb DNA-binding domain. Plant Physiol. 2000; 124: 491–493. 11027698 10.1104/pp.124.2.491 PMC1539279

[54] Zhou AL. Microarray analysis of rice grain abscission regulated by sh4. M.Sc. Thesis, Michigan State University. 2007.

[55] Dubos C, Stracke R, Grotewold E, Weisshaar B, Martin C, Lepiniec L. MYB transcription factors in Arabidopsis. Trends Plant Sci. 2010; 15: 573–81. doi: 10.1016/j.tplants.2010.06.005 20674465 10.1016/j.tplants.2010.06.005

[56] Baldoni E, Genga A, Cominelli E. Plant MYB transcription factors: Their role in drought response mechanisms. Int J Mol Sci. 2015; 16: 15811–15851. doi: 10.3390/ijms160715811 26184177 10.3390/ijms160715811 PMC4519927

[57] Himi E, Yamashita Y, Haruyama N, Yanagisawa T, Maekawa M, Taketa S. *Ant28* gene for proanthocyanidin synthesis encoding the *R2R3* MYB domain protein (Hvmyb10) highly affects grain dormancy in barley. Euphytica. 2012; 188: 141–151.

[58] Zhang YJ, Liang WQ, Shi JX, Xu J, Zhang DB. *MYB56* Encoding a R2R3 MYB Transcription Factor Regulates Seed Size in Arabidopsis thaliana. Journal of Integrative Plant Biol. 2013; 55: 1166–1178. 10.1111/jipb.12094 23911125

[59] Kaplan-Levy RN, Brewer PB, Quon T, Smyth DR. The trihelix family of transcription actors—light, stress and development. Trends Plant Sci. 2012; 17: 163–171. doi: 10.1016/j.tplants.2011.12.002 22236699 10.1016/j.tplants.2011.12.002

[60] Suzuki A, Suzuki T, Tanabe F, Toki S, Washida H, Wu CY, et al Cloning and expression of five myb-related genes from rice seed. Gene. 1997; 198: 393–398. 9370307 10.1016/s0378-1119(97)00344-2

[61] Katiyar A, Smita S, Lenka SK, Rajwanshi R, Chinnusamy V, Bansal KC. Genome-wide classification and expression analysis of MYB transcription factor families in rice and Arabidopsis. BMC Genomics. 2012; 13:544 doi: 10.1186/1471-2164-13-544 23050870 10.1186/1471-2164-13-544 PMC3542171

[62] Zhang F, Huang LY, Wang WS, Zhao XQ, Zhu LH, Fu BY, et al Genome-wide gene expression profiling of introgressed indica rice alleles associated with seedling cold tolerance improvement in a japonica rice background. BMC Genomics. 2012; 13:461 doi: 10.1186/1471-2164-13-461 22953761 10.1186/z PMC3526417

[63] Gao P, Bai X, Yang L, Lv DK, Li Y, Cai H, et al Over-expression of *osa-MIR396c* decreases salt and alkali stress tolerance. Planta. 2010; 231: 991–100. doi: 10.1007/s00425-010-1104-2 20135324 10.1007/s00425-010-1104-2

[64] Kyndt T, Denil S, Haegeman A, Trooskens G, Bauters L, Criekinge WV, et al Transcriptional reprogramming by root knot and migratory nematode infection in rice. New Phytologist. 2012; 196: 887–900. doi: 10.1111/j.1469-8137.2012.04311.x 22985291 10.1111/j.1469-8137.2012.04311.x

[65] Yokotani N, Sato Y, Tanabe S, Chujo T, Shimizu T, Okada K, et al *WRKY76* is a rice transcriptional repressor playing opposite roles in blast disease resistance and cold stress tolerance. J Experimental Botany. 2013; 64: 5085–5097. 10.1093/jxb/ert298 PMC3830488 24043853

[66] Kumari S, Shridhar S, Singh D, Priya P, Farmer R, Hundal J, et al The role of lectins and HD-ZIP transcription factors in Isoprenoid based plant stress responses. Proc Indian natn Sci Acad. 2012; 78: 671–691.

[67] Hwang SH, Kwon S, Jang JY, Fang L, Lee H, Choi C, et al *OsWRKY51*, a rice transcription factor, functions as a positive regulator in defense response against Xanthomonas oryzae pv. Oryzae. Plant Cell Rep. 2016; 35: 1975–1985. doi: 10.1007/s00299-016-2012-0 27300023 10.1007/s00299-016-2012-0

[68] Saha J, Sengupta A, Gupta K, Gupta B. Molecular phylogenetic study and expression analysis of ATP-binding cassette transporter gene family in *Oryza sativa* in response to salt stress. Comput Biol Chem. 2015; 54: 18–32. doi: 10.1016/j.compbiolchem.2014.11.005 25531538 10.1016/j.compbiolchem.2014.11.005

[69] Campos-soriano I, García-martínez L, Segundo BS. The arbuscular mycorrhizal symbiosis promotes the systemic induction of regulatory defence-related genes in rice leaves and confers resistance to pathogen infection. Mol Plant Pathology. 2012; 13: 579–592. 10.1111/j.1364-3703.2011.00773.x PMC6638712 22212404

[70] Ishimaru K, Hirotsu N, Madoka Y, Murakami N, Hara N, Onodera H, et al Loss of function of the IAA-glucose hydrolase gene *TGW6* enhances rice grain weight and increases yield. Nat Genet. 2013; 45: 707–711. doi: 10.1038/ng.2612 23583977 10.1038/ng.2612

