# Supplementary figures and images for: Transcriptome analysis of near-isogenic line provides novel insights into genes associated with panicle traits regulation in rice
Source: PLoS One. 2018 Jun 20;13(6):e0199077. doi: 10.1371/journal.pone.0199077 (PMC6010284; doi:10.1371/journal.pone.0199077)

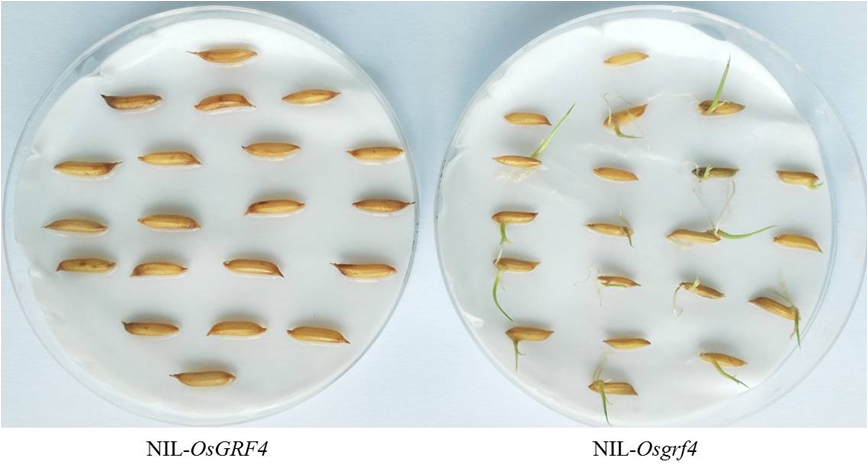

Supplement: S1 Fig — The seeds have been stored at room temperature over one year. (TIF) [file pone.0199077.s001.tif]

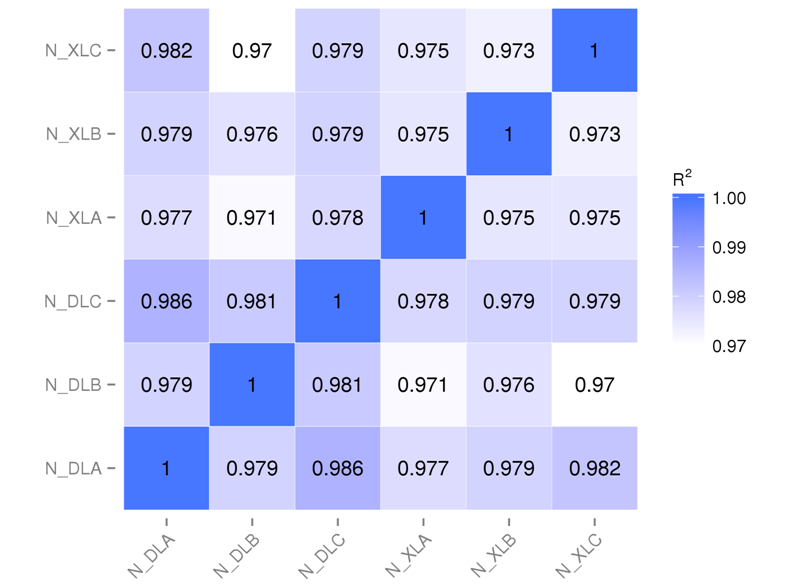

Supplement: S2 Fig — N_DL: NIL-OsGRF4; N_XL: NIL-Osgrf4; A, B and C represents three biological replicates. (TIF) [file pone.0199077.s002.tif]

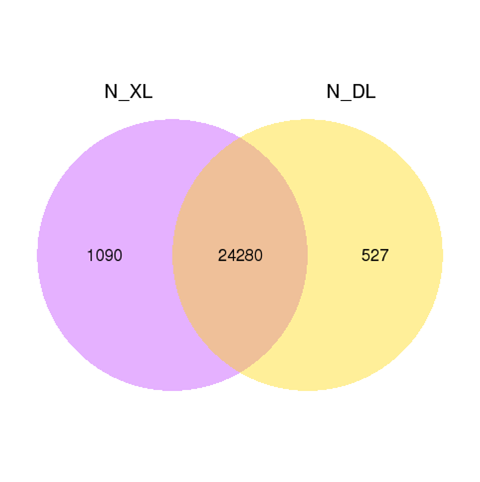

Supplement: S3 Fig — A total of 24807 and 25370 genes with expression were identified from NIL-OsGRF4 and NIL-Osgrf4, respectively. N_DL: NIL-OsGRF4; N_XL: NIL-Osgrf4. (TIF) [file pone.0199077.s003.tif]

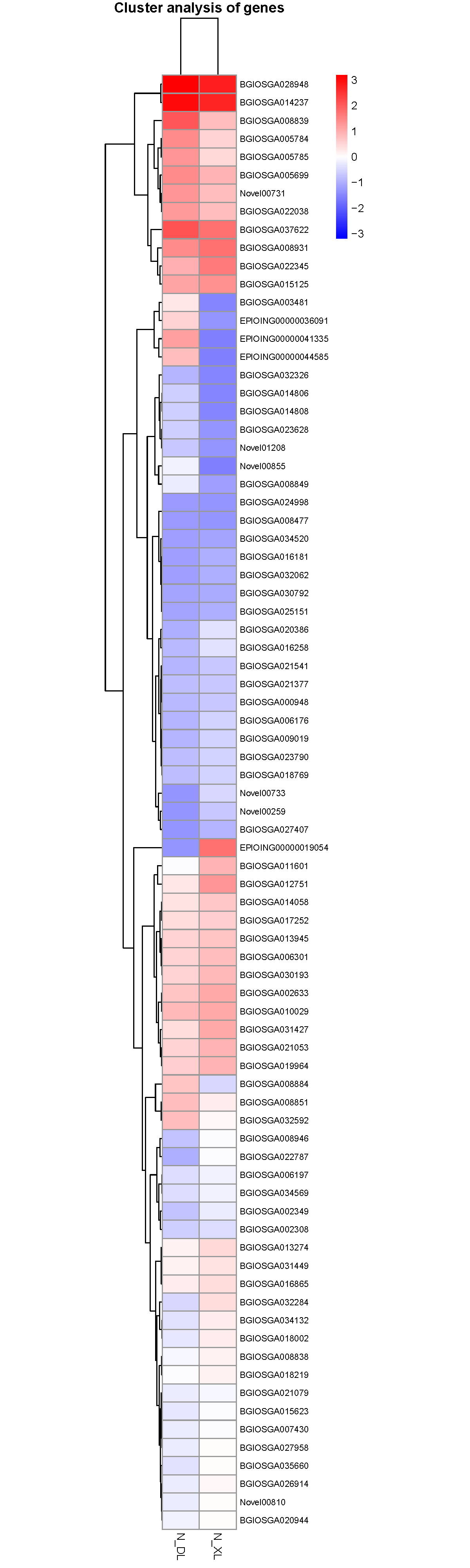

Supplement: S4 Fig — OsGRF4 (BGIOSGA005785) tightly clustered together with LOC_Os02g47320 (BGIOSGA005784). N_DL: NIL-OsGRF4; N_XL: NIL-Osgrf4. (TIF) [file pone.0199077.s004.tif]

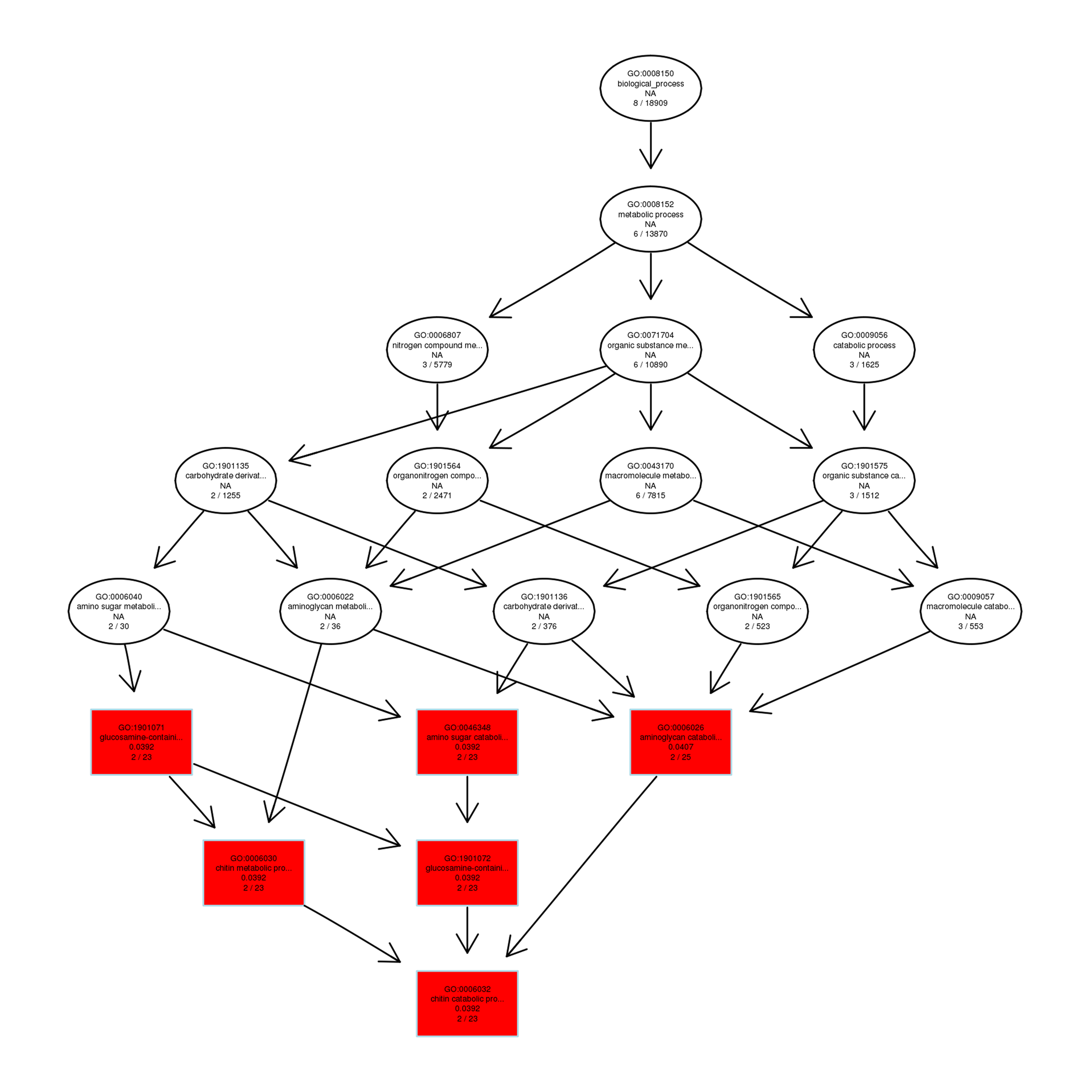

Supplement: S5 Fig — (TIF) [file pone.0199077.s005.tif]

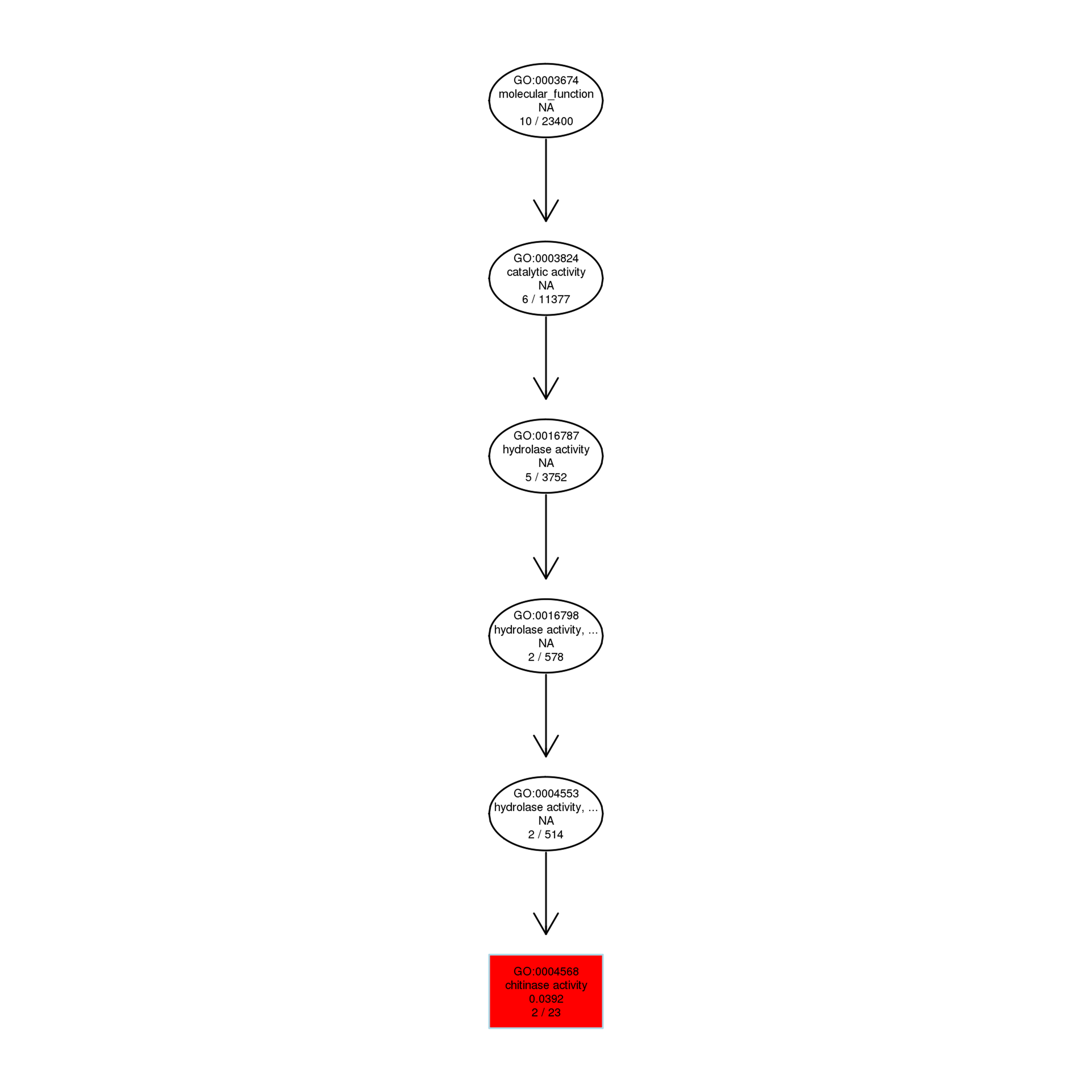

Supplement: S6 Fig — (TIF) [file pone.0199077.s006.tif]
